# The Mitochondrion-Like Organelle of *Trimastix pyriformis* Contains the Complete Glycine Cleavage System

**DOI:** 10.1371/journal.pone.0055417

**Published:** 2013-03-13

**Authors:** Zuzana Zubáčová, Lukáš Novák, Jitka Bublíková, Vojtěch Vacek, Jan Fousek, Jakub Rídl, Jan Tachezy, Pavel Doležal, Čestmír Vlček, Vladimír Hampl

**Affiliations:** 1 Charles University in Prague, Faculty of Science, Department of Parasitology, Prague, Czech Republic; 2 Institute of Molecular Genetics of the Academy of Sciences of the Czech Republic, Prague, Czech Republic; Université Joseph Fourier, France

## Abstract

All eukaryotic organisms contain mitochondria or organelles that evolved from the same endosymbiotic event like classical mitochondria. Organisms inhabiting low oxygen environments often contain mitochondrial derivates known as hydrogenosomes, mitosomes or neutrally as mitochondrion-like organelles. The detailed investigation has shown unexpected evolutionary plasticity in the biochemistry and protein composition of these organelles in various protists. We investigated the mitochondrion-like organelle in *Trimastix pyriformis,* a free-living member of one of the three lineages of anaerobic group Metamonada. Using 454 sequencing we have obtained 7 037 contigs from its transcriptome and on the basis of sequence homology and presence of N-terminal extensions we have selected contigs coding for proteins that putatively function in the organelle. Together with the results of a previous transcriptome survey, the list now consists of 23 proteins – mostly enzymes involved in amino acid metabolism, transporters and maturases of proteins and transporters of metabolites. We have no evidence of the production of ATP in the mitochondrion-like organelle of *Trimastix* but we have obtained experimental evidence for the presence of enzymes of the glycine cleavage system (GCS), which is part of amino acid metabolism. Using homologous antibody we have shown that H-protein of GCS localizes into vesicles in the cell of *Trimastix*. When overexpressed in yeast, H- and P-protein of GCS and cpn60 were transported into mitochondrion. In case of H-protein we have demonstrated that the first 16 amino acids are necessary for this transport. Glycine cleavage system is at the moment the only experimentally localized pathway in the mitochondrial derivate of *Trimastix pyriformis*.

## Introduction

In the last decades, systematic research has considerably improved our knowledge regarding the functions of mitochondrial homologues in many eukaryotic lineages. Particular interest has been paid to microbial parasites and protists that thrive facultatively or obligatorily under anaerobic or microaerophilic conditions (for recent reviews see e.g. [1]–[3]). It has been shown that their mitochondria often deviate remarkably from the text-book picture. For example, various reductions of components of membrane electron transport chain can be found. Instead of canonical four complexes plus F_o_F_1_ ATPase, the complexes III and IV are absent in *Blastocystis* and *Nyctotherus* derivates of mitochondrion 4–7. The path of electrons in these truncated electron transport chains ends at fumarate or in the case of *Blastocystis* also at oxygen to which the transfer is mediated by the complex of alternative oxidase [4]–[7]. Many obligatory anaerobes and microaerophiles lack a respiratory chain completely [3], [8], [9] and the enzyme [FeFe]hydrogenase provides the sink for electrons produced by redox reactions in their organelles. This enzyme transfers these electrons to protons producing hydrogen gas, a typical feature of hydrogenosomes that represent one functional class of organelles homologous to mitochondrion. Notable variation has evolved also in the enzymatic machinery metabolizing pyruvate. In mitochondria of anaerobes and microaerophiles, the canonical pyruvate dehydrogenase complex is usually substituted by the analogous enzymes pyruvate:ferredoxin oxidoreductase, pyruvate:NADH oxidoreductase or pyruvate formate lyase [10]–[12]. Some organisms possess two or even all three types of these enzymes. Finally, neither the metabolism of pyruvate nor the ATP production is a function common to all mitochondrial homologues. These processes are absent in the most minimalistic versions of these organelles – mitosomes of *Giardia, Entamoeba*, *Cryptosporidium* and microsporidia [8], [13]–[17]. Yet, the mitochondria even in their miniature form are apparently still essential for eukaryotic cells, as all eukaryotes studied so far possess them. The functions of these minimalistic mitochondrial homologues (mitosomes) and perhaps the most basic function of all mitochondrial homologues, has not been established yet. The synthesis of FeS clusters is often mentioned in this context [18].

Metamonada is a group composed exclusively of anaerobes and microaerophiles [19], [20]. The mitochondrial organelles of two metamonad lineages, parabasalids (i.e. *Trichomonas*) and fornicates (i.e. *Giardia*), have been extensively studied. It has been reported that the proteome of purified hydrogenosomes of *Trichomonas vaginalis* consists of more than 500 proteins, however, many of them may be only externally associated [9], [21]. The metabolism of the parabasalid hydrogenosome has been reconstructed to fine details and most enzymes have been biochemically characterized [22]. 139 proteins have been found in the mitosomal fraction of *Giardia*, however, only 20 of them have been experimentally verified as *bona fide* mitosomal proteins [8]. The only biochemically verified function of the *Giardia* mitosome remains the synthesis of FeS clusters [23]. The third lineage of Metamonada – Preaxostyla – consists of oxymonads and *Trimastix* [24]. Nothing is known about the mitochondrial homologues of oxymonads and besides one observation [25] no such organelles have been observed in this group. Double membrane bounded organelles have been described in *Trimastix* [26]–[28]. Several transcripts typical for mitochondrial proteins have been found among 10 000 transcriptome reads of *Trimastix pyriformis* (see Table 1 in [29]). Four of these transcripts (cpn60, H-protein, T-protein and P1-protein of glycine cleavage system) contained short extension at their 5' end in comparison with bacterial homologues, i.e. putative mitochondrial targeting sequences. However, none of these presequences are recognized by prediction software trained to recognize these sequences in other organisms. Likewise, none of these proteins have been experimentally localized to a cellular compartment. In this paper, we build on this previous work and present a more thorough transcriptome analysis based on 454 sequencing and more importantly bring the first experimental evidence for localization of cpn60 and enzymes of glycine cleavage system in the mitochondrial homologue of *Trimastix.*

**Table 1:** List of the proteins putatively localized in the mitochondrion-like organelle of *Trimastix pyriformis*.

Product	Sequence accession numbers	N-terminalextension	Experimental evidence
**Aconitase** TCA cycle enzyme	EU086483	Yes	No
**hydE** Maturation of [FeFe] hydrogenase	JX657285	Yes	No
hydF* Maturation of [FeFe] hydrogenase	JX657286	Yes	No
**hydG** Maturation of [FeFe] hydrogenase	JX657287	?	No
**H-protein of glycine cleavage system** central protein in GCS	EU086492	Yes	Yes
**P1-protein of GCS** Glycine dehydrogenase (decarboxylating) subunit 1	EU086490	Yes	Yes
**P2-protein of GCS** Glycine dehydrogenase (decarboxylating) subunit 2	EU086491	?	No
**L-protein of GCS** Dihydrolipoyl dehydrogenase	EU086501	No	No
**T-protein of GCS** Aminomethyltransferase	EU086485	Yes	No
**Lipoyltransferase** Lipoylisation of enzymes	EU086495	?	No
Serine hydroxymethyltransferase* Amino acid metabolism	JX657288	Yes	No
Ornithine transcarbamylase* Amino acid metabolism	JX657289	Yes	No
**Tom40** Protein transport	EU086500	NA	No
Sam50* Protein transport	JX657290	NA	No
Tim17 protein family member* Protein transport	JX657291	No	No
Pam18*Protein transport	JX657292	No	No
**Mitochondrial processing protease α subunit** Targeting sequence cleavage	EU086496	No	No
**Cpn60** Protein folding	EU086489	Yes	Yes
**Pyridine nucleotide transhydrogenase beta+alpha** NAD and NADP interconversion	EU086499	No	No
**Membrane carrier 1** Putative ATP/ADP transporter	EU086488	No	No
Membrane carrier 2* Putative 2-oxodicarboxylate carrier	JX657293	No	No
**Membrane carrier 3** Putative folate carrier	EU086487	?	No
Membrane carrier 4* Transporter with unknown specificity	JX657294	?	No

* The transcripts were identified in this study

## Results

### Proteins putatively localized to *Trimastix* mitochondrion-like organelle

In order to detect proteins putatively localized in the organelle of *Trimastix*, we have generated new set of transcriptomic data. In two runs of 454 sequencing of *Trimastix* mRNA we have produced in total 643 758 reads of *Trimastix* mRNA that were assembled into 7 037 contigs and 33 204 singletons. The contigs were automatically annotated using dCAS pipeline (http://exon.niaid.nih.gov). The contigs and singletons were then screened using HMM for proteins of protein transport machinery and mitochondrial carriers. Selected candidates were manually investigated for the presence of functional domains. Furthermore, the set of contigs and singletons was searched using standalone BLAST with *Giardia intestinalis* mitosomal proteins, *Trichomonas vaginalis* hydrogenosomal proteins and TCA cycle enzymes as queries. Best hits were further screened by predictor of protein localization Euk-mPloc 2.0. [30]. Putative organellar proteins predicted by Euk-mPloc, in which the presence of N-terminal targeting presequence is expected, were investigated for the presence of N-terminal targeting signal by three predictor programs (Table S1). Besides two exceptions (HydE and ornithine transcarbamylase), the proteins were not strongly predicted as mitochondrially targeted. Nevertheless, 9 proteins (including HydE and ornithine transcarbamylase) showed N-terminal extensions relative to the bacterial homologues in their alignments (Figure S1). Even if most of these extensions were not recognized as putative targeting peptides, we still consider this possibility and below present experimental evidence that the extension present in the H-protein of glycine cleavage system is indeed required for protein targeting into mitochondrion. We used the presence of an N-terminal extension as an important criterion for inclusion in the list of proteins predicted to localize into the mitochondrion-like organelle. Proteins in which the extension was not demonstrated were included only if they were functionally linked to other proteins in the list (hydG, P2-protein of glycine cleavage system) or if they were considered as strictly or almost strictly mitochondrially localized proteins (e.g. Tom40, Sam50, hydG, mitochondrial processing peptidase). The final list of proteins predicted to localize into the mitochondrion-like organelle of *Trimastix* on the basis of current data and the basis of data of Hampl et al. [29] is given in Table 1.

These proteins are involved in amino acid metabolism (glycine cleavage system, serine hydroxymethyltransferase, ornithine transcarbamylase), co-factor metabolism (pyridine nucleotide transhydrogenase β+α, lipoyltransferase), transport and maturation of proteins (Tom40, Sam50, one member of Tim17 family, Pam18, mitochondrial processing peptidase, cpn60 and [FeFe]hydrogenase maturases) and transport of other metabolites (proteins of membrane carrier family). Mitochondrial type aconitase is the only enzyme involved in energy metabolism that was included in the list. Enzymes of pyruvate:ferredoxin oxidoreductase (PFO) and [FeFe]hydrogenase were not listed because there are no strong indications that they are localized in the organelle. Neither [FeFe]hydrogenase nor PFO contained obvious N-terminal extensions. The substrate specificity of four identified membrane carriers (PFAM PF00153) was estimated according to the sequence similarity as well as to the presence of the residues known to be involved in the substrate binding [31]. Hence, the inner membrane of the mitochondrion-like organelle likely accommodates the ADP/ATP, 2-oxodicarboxylate and folate carriers. Although the fourth identified protein shares the signature motives of the protein family, the substrate specificity could not be estimated due to the high sequence divergence.

### Glycine cleavage system is localized to *Trimastix* mitochondrion-like organelle

Given the fact that the complete set of glycine cleavage system (GCS) enzymes has been found in the transcriptome of *Trimastix* and that three of these proteins (H-, P1- and T-protein) contained 5' extensions, it seem likely that the complete glycine cleavage system is localized in the organelle. To corroborate this hypothesis we have performed three experiments.

**Figure 1:**
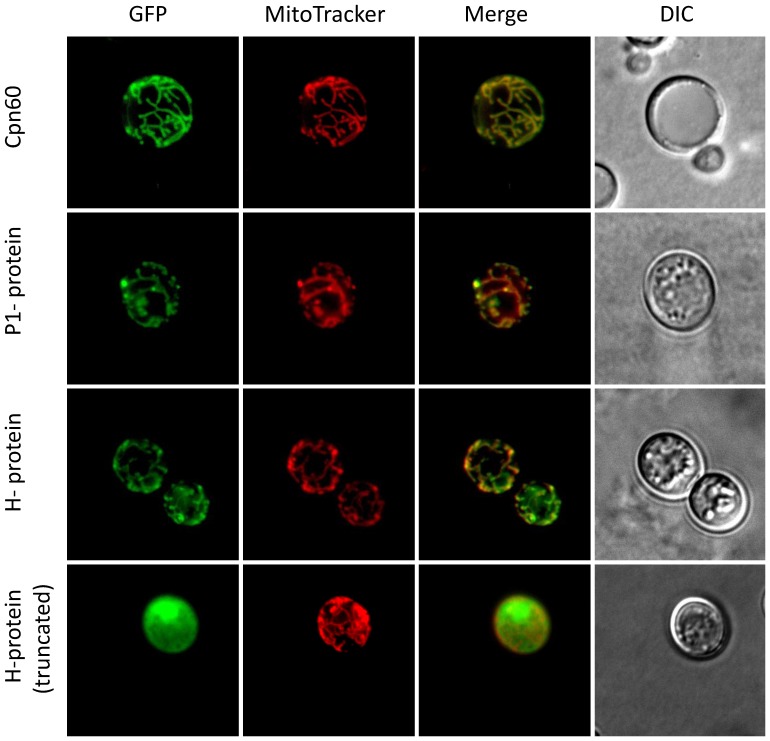
Over-expression of *Trimastix* proteins in yeast. The over-expression of GFP tagged proteins of *Trimastix* in *Saccharomyces cerevisiae*. The columns represent the signals from GFP tag (green), MitoTracker (red), merged GFP and MitoTracker and DIC. Rows represent individual proteins: cpn60, P1-protein of GCS, H-protein of GCS and H-protein of GCS truncated of the first 16 amino acids.

Firstly, we have used the *Saccharomyces cerevisiae* heterologous expression system with the assumption that protein localized into the mitochondrion-like organelles in *Trimastix* will also be recognized as mitochondrial protein by the yeast mitochondrion. As a positive control we have over-expressed a GFP-tagged version of *Trimastix* cpn60, the classical mitochondrial marker, in yeast. The fluorescence microscopy showed that the GFP signal co-localized with the signal from MitoTracker that highlighted yeast mitochondria (Figure 1). This demonstrates that the protein transport machinery of the yeast mitochondrion is able to recognize *Trimastix* organellar proteins. Analogously to cpn60, we over-expressed GFP-tagged P1- and H-proteins. The fluorescence microscopy showed that the GFP signal co-localized with the signal from MitoTracker (Figure 1) indicating that both proteins were transported into the yeast mitochondria. As a negative control we have over-expressed a GFP tagged H-protein that was truncated at the N-terminus and started with the 17^th^ amino acid. The truncated H-protein remained in the cytosol of yeast (Figure 1). Besides serving as a negative control, the latter experiment also confirmed our expectation that the N-terminal extension observed in H-protein bears a signal necessary for targeting of the protein into the mitochondrion-like organelle.

**Figure 2:**
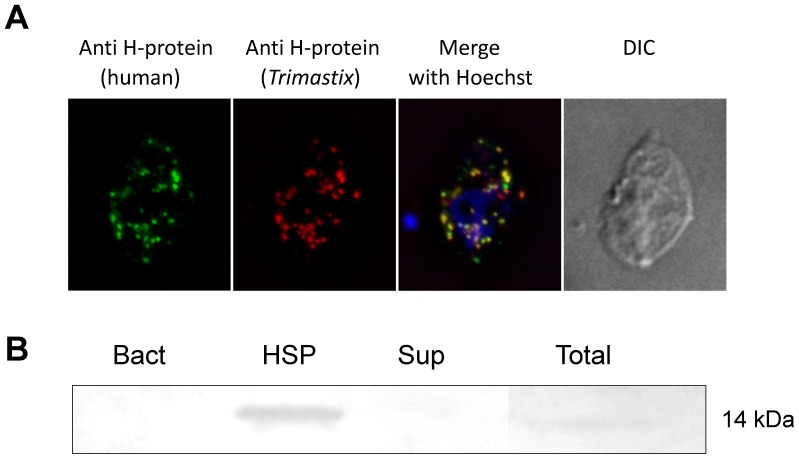
H-protein of GCS localizes into vesicles (putative mitochondrion-like organelles) in *Trimastix pyriformis*. A) Immunofluorescence microscopy of the *Trimastix pyriformis* cell. The green signal from antiH-protein (human) co-localizes with red signal from the antiH-protein (*Trimastix*). The DNA is stained blue with Hoechst. B) Western blot on the cellular fractions of *Trimastix pyriformis.* The lines represent pure bacteria *Citrobacter* sp. from the culture (Bact), high speed pellet of *Trimastix* (HSP), supernatant of *Trimastix* (Sup), total lysate of *Trimastix* (Total).

Secondly, we have used immunofluorescence microscopy (Figure 2A, Figure S2) with two antibodies against the H-protein of GCS – a commercial antibody against human H-protein (green signal) and our in-house prepared antibody against *Trimastix* H-protein (red signal). The green signal showed several spots that co-localized with the red signal revealing dozens of bodies (putative mitochondrion-like organelles) distributed predominantly around the nucleus and in the posterior-ventral part of the cell.

Finally, we have applied the antibody against *Trimastix* H-protein on the Western blot of cell fractions of *Trimastix* (Figure 2B). The signal of the expected size appeared in the high speed pellet (HSP) and in the total lysate of *Trimastix* but neither in the lysate of bacteria from the *Trimastix* culture (Bact) nor in the supernatant (Sup) that contains the cytoplasm of *Trimastix*.

To see whether the organelle of *Trimastix* produces observable proton potential, we have stained the cell of *Trimastix* with MitoTracker Red CMXRos dye that specifically accumulates in the mitochondria upon the presence of the membrane potential. No stained vesicles were observed in *Trimastix* cells and only diffused cytosolic signal was detected. Similar results were obtained when MitoTracker Green FM was used, which does not require membrane potential (not shown).

## Discussion

In this second transcriptomic study of *Trimastix pyriformis* we have produced, using 454 technology, more than 60x more reads which formed 2,6x more contigs (not counting singletons) than in the previous study [29]. Despite the massive increase in the amount of data, we were able to predict only 8 new proteins that putatively localize to the mitochondrion-like organelle (marked by stars in the Table 1). These include HydF, serine hydroxymethyltransferase, ornithine transcarbamylase, Sam50, Tim17 protein family member and Pam18. The number of contigs assembled (7 037 in this data set) is unlikely to cover the complete transcriptome and so the discovery of new organellar proteins is expected in the future.

In addition to the *in silico* study, we gathered the first experimental evidence in support of organellar localization of cpn60 and two of the four enzymes of glycine cleavage system (H- and P1-protein). The evidence for putative functions of the mitochondrion-like organelle is discussed below.

### Amino acid metabolism

As many as seven enzymes in the list are directly involved in amino acid metabolism, namely H-, P1-, P2-, T- and L-protein of GCS, serine hydroxymethyltransferase (SHMT) and ornithine transcarbamylase (OTC), the eighth enzyme, lipoyltransferase, is involved only indirectly by lipoylisation of the H-protein [32].

The GCS catalyses a cycle of glycine catabolising reactions producing methyl-tetrahydrofolate, NADH and CO_2_ and it can function also in the opposite direction [33]. In eukaryotes, the cycle is typically localized in the mitochondrion. The evidence for the localization of GCS in the mitochondrion-like organelle of *Trimastix pyriformis* seems to be relatively strong. All five enzymes are present in the transcriptome (the two subunits of P-protein are coded as separate proteins). Three of them (H, P1 and T) carry an N-terminal extension and in the case of H-protein we have shown that the N-terminal extension is necessary for its targeting to the yeast mitochondrion. Two of these proteins (H and P1) have been transported into the mitochondrion when over-expressed in yeast, and finally the H-protein has been shown to be present in vesicles (putative mitochondrion-like organelles) in *Trimastix*, by co-localization of two antibodies. Although the ultimate evidence of immunoelectron microscopy of *Trimastix* with anti H-protein antibodies is still missing, considering the fact that GCS has never been observed outside mitochondria or relative organelles in other eukaryotes, the presence of the pathway in the mitochondrion-like organelle of *Trimastix* is very likely.

Serine hydroxymethyltransferase catalyses a reversible conversion of L-serine and tetrahydrofolate to glycine and 5,10-methylenetetrahydrofolate. The reaction may therefore be directly connected to GCS. Various isoforms of SHMT are present in the cytosol, mitochondria and plastids of eukaryotes [34]. The *Trimastix* enzyme contains an N-terminal extension when compared to the bacterial counterparts and so we regard it as putatively localized into the mitochondrion-like organelle (Figure S1).

Ornithine transcarbamylase catalyses the reaction between ornithine and carbamoyl phosphate with the formation of citrulline. This reaction is a part of arginine catabolism in some protists (arginine dihydrolase pathway) and of the urea cycle in mammals. The arginine dihydrolase pathway consists of three enzymes: arginine deiminase (ADI), OTC and carbamoyl kinase (CK). It is localized in the hydrogenosome of *Neocallimastix frontalis* [35] but in the cytosol of *Giardia* [36], where it represents an important source of ATP. In *Trichomonas vaginalis*, the pathway is believed to be present also in the cytosol, however one enzyme of the pathway, ADI, was found in the hydrogenosome [37]. While ADI was not found in the transcriptome, CK is likely present in *Trimastix pyriformis*. Similar to OTC, the *Trimastix* CK is related to prokaryotic CKs but unlike OTC it apparently does not carry an N-terminal extension and therefore was not included in the Table 1. The prokaryotic nature of both enzymes suggests that they may represent bacterial contamination of the transcriptome data set. On the other hand, the relatively high number of reads for these transcripts (1486 for OTC and 640 for CK), which is more than the number of reads of H-protein of GCS (233 reads) or SHMT (210 reads) indicate that they may represent *bona fide Trimastix* enzymes. The prokaryotic origin of *Trimastix* enzymes is, in fact, quite common and other examples of such enzymes are the P1-protein of GCS [29], for which organellar localization was confirmed experimentally in this paper, and 4 out of 10 glycolytic enzymes [38]. The confirmation of the presence and cellular localization of arginine dihydrolase pathway in *Trimastix pyriformis* deserves future research.

### Energy metabolism

The only protein in the list directly involved in the energy metabolism is a tricarboxylic-acid-cycle-enzyme aconitase. The localization of a sole enzyme from the cycle in the compartment is, however, very suspicious, and this localization must be verified experimentally before it should be considered more seriously. Even if its localization was confirmed the actual function of the solitary enzyme would remain questionable. Nevertheless this protein fulfills the conditions to be included in the list. Being a homologue of mitochondrial type aconitase and not the cytosolic version it was predicted to localize in the mitochondrion-like organelle by Euk-mPloc 2.0. and, furthermore, it contains a short N-terminal extension.

The set of all three maturases of [FeFe]hydrogenase was found in the transcriptome. Contigs for two of them have complete N-terminus with an extension. These enzymes are essential for maturation of [FeFe]hydrogenase in bacteria [39] but they have been reported from only 5 eukaryotes so far: *Trichomonas vaginalis*, *Chlamydomonas reinhardtii*, *Mastigamoeba balamuthi*, *Acanthamoeba castelanii* and *Andalucia incarcerate* [10], [40], [41]. In *Trichomonas* and *Chlamydomonas* these proteins are localized in the hydrogenosomes and plastids respectively [40], [41]. It is generally believed that the maturases are always localized in the organelle where they assist the maturation of the H-cluster of [FeFe]hydrogenase. The presence of the N-terminal extensions makes them serious candidates for organellar proteins in *Trimastix*. The presence of maturases would suggest that the [FeFe]hydrogenase itself is present in the organelle as well. So far we have no evidence for the localization of [FeFe]hydrogenase and none of the three homologues present among the transcripts bears N-terminal extension indicating the organellar localization. For this reason, [FeFe]hydrogenase was not included in the Table 1. The same applies to pyruvate:ferredoxin oxidoreductase, an enzyme that is often functionally connected to [FeFe]hydrogenase.

### Protein transport

**Figure 3:**
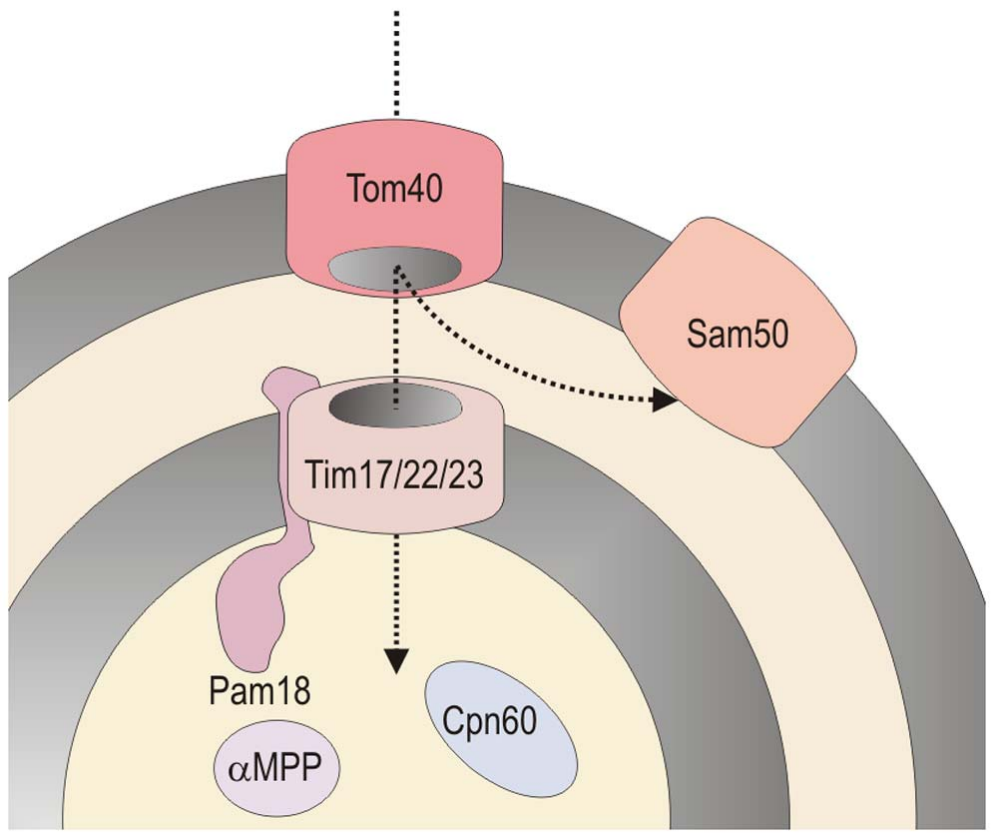
Schematic representation of protein import machinery in *Trimastix pyriformis* mitochondrion-like organelle.

Six proteins involved in the transport, processing and maturation of proteins (not counting the specific [FeFe]hydrogenase maturases) have been found: Tom40, Sam50, one member of Tim17/22/23 family, Pam18, α subunit of mitochondrial processing peptidase (αMPP) and cpn60 (Figure 3). This set of proteins represents the basic functional core of protein transport machinery: Tom40 and Tim17/22/23 being the outer- and inner-membrane transport pores, respectively, Sam50 functions as assembly machinery for Tom40 and Pam18 being the part of the motor complex associated with Tim17/22/23 translocase. Upon protein import the MPP cleaves off the targeting peptides and cpn60 assists the protein folding. The *Trimastix* protein transport machinery in this composition would be slightly more complex than the machinery in the mitosome of *Giardia* where the inner membrane pore and Sam50 is missing [8]. We however expect that the *Trimastix* protein transport machinery set is not complete yet and more components will be discovered in the future. Conspicuously absent from all *Trimastix* genomic data sets are the genes encoding βMPP and mtHsp70, two proteins that have been found in most mitochondrion-related organelles examined to date.

### Other membrane proteins

Pyridine nucleotide transhydrogenase (PNT) used to be regarded as a specific protein of the inner membrane of the mitochondrion [42] until Yousuf et al. [43] have shown that it localizes into vesicles different from mitosomes in *Entamoeba histolytica.* PNT transfers hydride ion between NAD(H) and NADP(H) and simultaneously transfers proton across the membrane [44]. Structurally the protein functions as a homodimer and each monomer consist of two domains α and β. These domains are expressed as separate proteins in prokaryotes but as a single protein in eukaryotes. In the first study of the transcriptome of *Trimastix* [29], we found the domains in separate contigs and concluded that they were expressed independently as in prokaryotes. In the assembly of the 454 reads, however, the two subunits appeared in a single contig suggesting that the two domains are encoded by a single gene and expressed as a single protein like in other eukaryotes.

In the present and previous study we have identified altogether four members of the mitochondrial carrier family and we designated them as membrane carrier protein 1–4. As proteins from this family have also been reported from the membranes of peroxisomes and plastids [45]–[47], their presence in the membrane of mitochondrion-like organelle is only putative. The carriers designated now as carriers 1 and 3 have been previously reported upon [29], the carriers 2 and 4 were identified in the current data set. The carrier 3 listed in the Table 1 in [29] has been excluded from the current list, as we have serious doubts about its affiliation into mitochondrial carrier family. According to the conserved residues and phylogenetic relationships to other carriers we expect that carrier 1 transports adenine nucleotides (e.g. ATP, NAD), carrier 2 transports 2-oxodicarboxylates (e.g. 2-oxoglutarate) and carrier 3 transports folate. The substrate specificity of carrier 4 cannot be predicted from the sequence itself. The presence of glycine cleavage complex in the organelle indeed requires the transport of NAD/NADH and folate but also the transport of amino acids (glycine or serine). The latter molecules may be transported by carrier 4 or by carriers that have not been identified so far. Mitochondrial carriers typically need a proton potential across the inner mitochondrial membrane to properly function [31]. As we were not able to detect a proton potential using MitoTracker Red, it is possible that the carriers can operate under small or even without membrane potential. Similarly the carrier proteins of peroxisomes [31] and *Entamoeba* mitosomes [48] are thought to be membrane potential-independent.

## Conclusions

The transcriptome sequencing using 454 technology enriched the list of proteins putatively localized into the mitochondrion-like organelle of *Trimastix* to a total number of 23 proteins. Most of these proteins are involved in the metabolism of amino acids, transport and maturation of proteins and transport of metabolites. Neither PFO nor [FeFe]hydrogenase were included in the list as there is no evidence for them to be present in the organelle neither there is evidence that the organelle produces ATP. Mitochondrial localization of most of the listed proteins remains only putative and should be confirmed experimentally in the future. The first such evidence has been presented for the enzymes of glycine cleavage complex, which is at the moment the only experimentally localized pathway in the *Trimastix* mitochondrion-like organelle.

## Materials and Methods

### Preparation of *T*. *pyriformis* cDNA

*T. pyriformis* (strain RCP-MX, ATCC 50935) total RNA was isolated from 16×10^7^ cells using TRIzol Reagent (Invitrogen). *T. pyriformis* mRNA transcriptome was captured from total RNA with Dynabeads mRNA Purification Kit (Invitrogen). cDNA was then prepared using Smarter PCR cDNA Synthesis Kit (Clontech) according to the manufacturers protocol with 19 cycles of cDNA amplification.

### 454 transcriptome sequencing and annotation

Sequencing library optimized for Roche/454 Titanium sequencing was prepared using GS FLX Titanium Rapid Library Preparation Kit from double-stranded cDNA. Fragment library was titrated by enrichment and prepared for sequencing by emulsion PCR on two regions of a two-region GS-FLX Titanium PicoTitreTM plate. The reads were cleaned of all adaptor/primer and polyA sequence. Newbler (v2.6; Roche/454 Sequencing) and the default parameters (40 bp overlap; 90% identity) were used for the assembly of 644 537 reads (average length 399 bp). These were assembled into 7 037 contigs and 6 255 isogroups (33 204 singletons remained). Isogroups can either represent alternatively spliced genes (with contigs indicating exons, and isotigs representing splice forms), or sets of recently duplicated genes (with contigs representing regions of divergence since duplication, and isotigs representing the divergent genes) either as gene families or multiple alleles of the same gene.

All contigs were automatically annotated using dCAS pipeline (http://exon.niaid.nih.gov). In this pipeline all the contigs were analyzed by SignalP 3.0 server [49] to predict import signals and with TMHMM2.0 server [50] to predict transmembrane α-helices. Local BLASTX search against downloaded NCBI database (non redundant protein database from 11.7.2012) was used for annotation of contigs.

Candidate proteins of membrane protein translocation complexes were determined by HMM search of all six frame translation of contigs and singletons. The selected transcripts were further analyzed by HHpred search at http://toolkit.tuebingen.mpg.de/hhpred [51].

Standalone BLAST searches against the *Trimastix* contigs and singletons were performed in BioEdit 7.1.3.0. [52] using the set of 20 mitosomal proteins of *Giardia intestinalis* [8] and 413 hydrogenosomal proteins of *Trichomonas vaginalis* (Table S1 in [9]) as queries. The best hits were further submitted to Euk-mPloc 2.0 [30] for prediction of cellular localization. Proteins that were predicted to localize into mitochondria or chloroplasts were further investigated. For each such candidate for mitochondrial matrix protein, 10–20 closest eukaryotic and prokaryotic homologues were downloaded from the GenBank. The proteins were aligned and the alignment was manually refined in BioEdit 7.1.3.0. [52]. The completeness of the *Trimastix* protein sequences, the start codons and the presence or absence of N-terminal extension were estimated based on this alignment. *Trimastix* proteins that exhibited N-terminal extension relatively to the prokaryotic homologues were selected.

The sequences of newly determined candidate organellar proteins are stored in GenBank under accession numbers JX657285-JX657294. The Transcriptome Shotgun Assembly project has been deposited at DDBJ/EMBL/GenBank under the accession GAFH00000000. The version described in this paper is the first version, GAFH01000000.

### Preparation of constructs for over-expression in yeast

*T. pyriformis* genes were PCR amplified from cDNA using EmeraldAmp Max PCR Mastermix (Takara) and the following primers: Glycine cleavage system H-protein (GenBank ID: EU086492) – **5`TCTAGA**ATGCAGCGCCTTTTCTCT (XbaI site in bold) and **5`AAGCTT**ATGCTGGGTCTTGAGGAA (HindIII site in bold); N-terminally truncated version of H-protein – **5`TCTAGA**ATGGCTCGGTTTGCCGGCGAG (XbaI site in bold) and **5`AAGCTT**ATGCTGGGTCTTGAGGAA (HindIII site in bold); P1 protein of glycine cleavage system (GenBank ID: EU086490) – **5`TCTAGA**ATGCAGAACCTTTCTCGC (XbaI site in bold) and **5`AGCTT**CAGGGAGGCGCGCAGGGC (HindIII site in bold); cpn60 (GenBank ID: EU086489) – **5`TCTAGA**ATGCAGGCCCTGTTTTCC (XbaI site in bold) and **5` AAGCTT** GAATGGCTTGGGCAGGCC (HindIII site in bold). The PCR products were cloned into pUG35 vector with GFP tag at the 3` end.

### Transformation of yeasts

The wild type *Saccharomyces cerevisiae* strain YPH499 (ATCC number: 204679) was used in this study. Yeasts were grown on plates with YPD agar medium (for 500 ml: D-glucose, Penta: 10 g; yeast extract, Oxoid: 5 g; trypticase peptone, BBL: 10 g; agar, Oxoid: 6 g) at 30°C. Transformation of the yeasts with 2 µg of plasmid DNA was performed using LiAc/SS-DNA/PEG method according to Gietz and Schiestl [53]. Transformants were selected on synthetic drop-out medium without uracil (for 500 ml: D-glucose, Penta: 10 g; yeast nitrogen base, Sigma: 3,35 g; yeast synthetic drop-out medium supplement, Sigma: 0,96 g; agar, Oxoid: 6 g) at 30°C. Only transformants containing plasmids with cloned *T. pyriformis* genes were able to grow on medium lacking uracil. Expression of GFP-tagged proteins of *T. pyriformis* in yeasts was analyzed 3 days after transformation. Mitochondria were labeled with MitoTracker Red CMXRos dye (Molecular probes, cat. # M7512).

### Antibody production

Rat polyclonal antibody was raised against *T. pyriformis* GCS H-protein. A 6xHis-tagged version of this protein was expressed from plasmid pET42b in *Escherichia coli* BL21 DE3. Protein was purified by immobilized-metal affinity chromatography using Ni-NTA resin under denaturing conditions using 8 M urea according to the protocol described in the QIAExpressionist handbook (Qiagen). A rat was immunized with purified protein in acrylamide gel for a period of 12 weeks (300 µg of antigen was used per 1 subcutaneous injection every 4 weeks).

The serum specific for *T. pyriformis* GCS H-protein was tested for reactivity on Western blot using *Trimastix* cell fractions (whole cell lysate, cytoplasm, high speed pellet) as well as *Citrobacter* sp. lysate.

### *Trimastix* fractionation of cellular extracts

*T. pyriformis* cell fractions (cytosol and organelle-rich fraction) were obtained by differential centrifugation as previously described [54] with slight modifications. *T. pyriformis* (2.5 liters of the cell culture) was filtered from bacteria using Cyclopore Track Etched Membrane, 3µm (Whatman). Filtered *Trimastix* was pelleted by centrifugation for 10 minutes at 3000 x g. Cells were resuspended in 1 ml of cold 3% LB medium (L3022, Sigma; for 3% LB dilute 30 ml of LB medium in 970 ml of distilled water) containing protease inhibitor cocktail (Roche, cat. # 11836170001). Cells were placed on ice and homogenized by sonication (1–2 times for 1 minute at amplitude 40). Cells were checked by light microscope after each round of sonication. Homogenate was centrifuged for 10 minutes at 500 x g at 4°C. The pellet was discarded. The supernatant was centrifuged 30 minutes at 100000 x g at 4°C to pellet the organelles. Organelles were resuspended to final volume of 50µl of 3% LB medium containing protease inhibitor cocktail. The supernatant containing the cytosol was centrifuged again for 45 minutes at 100000 x g at 4°C. The pellet was discarded.

*T. pyriformis* cell fractions were analyzed by SDS-PAGE and Western blotting.

### Preparation on *Trimastix pyriformis* immunofluorescence slides

The slides were prepared using immunostaining protocol with coverslips according to Dawson et al. [55] with the following modifications. *Trimastix* cells in the growth medium were fixed with 2% paraformaldehyde solution for 30 minutes at room temperature. Fixed cells were dispensed on coverslips coated with 15 µl of Poly-L-lysine solution (Sigma) and left for one hour to adhere. Coverslips with adhered cells were air dried. Preparations were blocked with PEMBALG solution (PEM buffer; 1% BSA; 0,5% cold water fish skin gelatin; 100 mM lysine; 0,1% sodium azide) for 30 minutes at room temperature. Cells were incubated overnight with antibodies against human GCS H (Abnova) and against *Trimastix* GCS H (both diluted 1∶200) on parafilm. Preparations were incubated on parafilm with secondary antibodies AlexaFluor 488 Goat Anti-Mouse and AlexaFluor 594 Goat Anti-Rabbit (Molecular probes) diluted 1∶1000. Coverslips were washed three times with PEM buffer. The last wash was performed with addition of the Hoechst 33342 stain (Molecular probes) into PEM buffer (1∶1000 of dilution). Coverslips were mounted onto slides using VECTASHIELD Mounting Medium (Vector Laboratories).

### Immunofluorescence microscopy

The images were collected using a fluorescence microscope IX81 equipped with IX2-UCB camera (Olympus) with a 100x immersion oil objective and CelĺR software. Images were processed by ImageJ software (NIH, Bethesda, MD, USA).

## Supporting Information

Figure S1 **The N-terminal parts of protein alignments demonstrating the presence of extension in** ***Trimastix*** **protein relatively to the prokaryotic homologues.**
(PDF)

Figure S2 **Immunofluorescence microscopy of two additional** ***Trimastix pyriformis*** **cells.** The green signal from antiH-protein (human) co-localizes with red signal from the antiH-protein (*Trimastix*). The DNA is stained blue with Hoechst.
(PDF)

Table S1 **The probability of mitochondrial localization of selected** ***Trimastix*** **proteins as predicted by PSORT II, TargetP and Multiloc2 programs.**
(DOCX)
