# Supplementary material for: The Mitochondrion-Like Organelle of Trimastix pyriformis Contains the Complete Glycine Cleavage System
Source: PLoS One. 2013 Mar 13;8(3):e55417. doi: 10.1371/journal.pone.0055417 (PMC3596361; doi:10.1371/journal.pone.0055417)
Supplement: Figure S1 — The N-terminal parts of protein alignments demonstrating the presence of extension in Trimastix protein relatively to the prokaryotic homologues. (PDF) [file pone.0055417.s001.pdf]

**Figure S1.** The N-terminal parts of protein alignments demonstrating the presence of extension in *Trimastix* protein relatively to the prokaryotic homologues.

## Aconitase

```
Trimastix          -----------------------------MSTPLLQTPLSLYDQTPL-----PYERQLAKLAQVRAKLGRQDLTLAEKVLFTHVADVEALS--TAPGTYL
Nasonia            MSYCTRILQAQKVASLAAEVQQRCFSTSPLTFAAAKVAMSKFDSTAY----LPYDKLDENIKIVK-KRLDRPLTLSEKILYSHL-DEPQKQDIVRGTSYL
Dictyostelium      -----------------MNSLVKGISKVRSTRSFSTVSMSPLEPNKK----LNYEGIDAKLKQFR-LHHNKPLTLAEKIIYGHL-EDPSTK-VERGITYL
Monosiga           -----------MLRVNASRAALVGVRAFGTSRVAAASVPMSRFDDAP----MPYDTMRKRLDIVR-GRLNRPLTLSEKVLYSHL-DEPETQDIERGVSYL
Phytophthora       ----------------------------MSSSCGVPVPLSVLDPKGSPTIDQTYEKMEKNLYTVRKTNGNKPLTLAEKIIYGHL-DDATTK-AKRGETYL
Ignavibacterium    -------------------------------------MTANFDMIKK-----VYSSYKQKLADAR-KVLNRPMTYAEKVLYTHLWEKP-TREYVRGKDYV
Melioribacter      -------------------------------------MAANFDMIKR-----VYDNFENKVDKAR-KVVGRPLTYAEKVLYAHLAEEP-TREFVRGKDFV
Leptospira         -------------------------------------MAFDIEMIRA-----RYAKIGDLVTKAR-NVVGRPLTLTEKILYSHLWEGEPKAAYEKGKSYV
Flavobacteria      -------------------------------------MAFDIEMIEK-----VYANMVARVDKAR-ELVGKPLTLTEKILYSHLWDETSTNVYKRGVDYV
Spirosoma          -------------------------------------MAFDLDMIQR-----VYANLGERVEAAR-KAVGKPLTLSEKILYSHLFAGTPTQALERGKDYV
```

## HydE

```
Trimastix              --------------------------------------------------------------MRSTPIPDGVSDRVRKIWNFGGENSLSKRPESEAKAIP
Chlamydomonas          MAHSLSAHSRQAGDRKLGAGAASSRPSCPSRRIVRVAAHASASKATPDVPVDDLPPAHARAAVAAANRRARAMASAEAAAETLGDFLGLGKGGLSPGATA
Trichomonas            --------------------------------------------------------------------------MHNTLVPTSRHLPSFSEALEVAISGK
Trichomonas            -------------------------------------------------------------------------MQLNTLNPTTRELPSFSEALKVAVAGE
Caldicellulosiruptor   ---------------------------------------------------------------------------------MREEKMKVKEILDKAYNEH
Ruminococcus           ----------------------------------------------------------------------------------------MKRLIDKLHHEQ
Thermotoga             ---------------------------------------------------------------------------------------MTGREILEKLERR
Thermoanaerobacter     --------------------------------------------------------------------------------------MKVKEILEKAYHES
Symbiobacterium        -----------------------------------------------------------------------------------MIRQEVLALIARAEAGA
Clostridium            --------------------------------------------------------------------------------------MKIKDIIDKAYVES

Trimastix              DLSFDELKMLLQ----KHTPETDAALFRYSNSIANQLFGNKCYVRALVEFSNVCRKNCKYCGIRKDMKGVRRYTIPDHELMALAKFAYENKFTSLVLQSG
Chlamydomonas          NLDREQVLGVLEAVWRRGDLNLERALYSHANAVTNKYCGGGVYYRGLVEFSNICQNDCSYCGIRNNQKEVWRYTMPVEEVVEVAKWALENGIRNIMLQGG
Trichomonas            KMSHDHIVRLLN----AREKSEVDALHEAAGEVTKRNFHNEVSVRGIVEFSNACEKNCHYCGVNSYEP----FVIPHEAILDCCEFMWKKGYRNLVLQSG
Trichomonas            KMSHDHIVRLLN----PRTKDEIDALHSAAGAVTQRVFHNEVSVRGIVEFSNACEKRCHYCGVNSYEP----FLIPQEAILNSCKFMWAKGYRNLVLQSG
Caldicellulosiruptor   HLTKDEIKFLLN----SEGEEK-ELLFEYADRVRKEYVGDDVYLRGLIEFSSYCKNDCFYCGLRRSNKEAQRYRMQEDEIVEVAKRAYDMGYRTVVLQSG
Ruminococcus           MLTAEEFHRLLA----NRSEETDQYARELANQVRQEVYGNKIYVRGLIEFTNYCKNDCYYCGIRRSNQNAQRYRLTEEDILECCRQGYEIGFRTFVLQGG
Thermotoga             EFTREVLKEALS----INDRGFNEALFKLADEIRRKYVGDEVHIRAIIEFSNVCRKNCLYCGLRRDNKNLKRYRMTPEEIVERARLAVQFGAKTIVLQSG
Thermoanaerobacter     NLTKDEIKLLLM----AEGEDK-DLLFKTADRVRKEHVGDEVFLRGLIEFSSYCKNDCFYCGLRRSNQNAQRYRMQEDEIVEVAKRAYQMGYRTVVLQSG
Symbiobacterium        DLSRDELTCLLS----LGGEEQ-KALFAAADRVRARVMGDGVHLRGLIEFSNICRMDCCYCGIRRSNRHVRRYRMTPEEIVAAAQGAWRLGYGSVVLQSG
Clostridium            NLSQEEIVEILK----NKDEYNIKYLFNKAEETTEKYCGHEVNIRGIIEFSNYCRCNCSYCGLNVNNNGIKRYRMSKEEIVLVAKEAYEAGYKTLVLQSG
```

## HydF

```
Trimastix            MLSRSLLLRGSLLDSLQYMPFSAAASTTVQKPLPFFSKLFGLRTPHAAPSDLSLEPARAAGPGSELQLRTNIGIFGRMNAGKSTLINAITQQNTSIVDAH
Trichomonas          ---------------------------------------------------MLTSIGRYFAKKGNDLPRTHISMVGFMNAGKSTIMNAITQQPTSIVDST
Carboxydibrachium    ------------------------------------------------------------MNTTPTASRLHIGIFGRRNAGKSSLINALTNQEIAIVSDV
Desulfotomaculum     ------------------------------------------------------------MENTPRGNRLHIAIFGRRNAGKSSLINALTSQDIAVVSNI
Dorea                ----------------------------------------------------------MSLNSTPSSERIHIGIFGRRNAGKSSLINALTGQNLAIVSDV
Thiorhodococcus      ------------------------------------------------------------MLDTPKSLRLHIGIFGRRNAGKSSLINALLGYPLSIVSDV
Halanaerobium        ------------------------------------------------------------MNSTAKGDRPHIAVFGRRNVGKSSLINRLTNQDLALVSSQ
Desulfotalea         ---------------------------------------------------------MVFMQTVPKSMRLHLGIFGRTNVGKSTFLNFLVDQDVAIASSQ
```

## Serine hydroxymethyltransferase

```
Trimastix        ---------------------------MLASAISVPATALVAHASTGNIPLKEADPELYEIIEKERKRQSDGLELIASENFVSRAVLDCLGSCMTNKYSE
Zea mays         MAMATALRKLSANALRRQPLSRITPLYYMASLPATEERSGITWTKQLNAPLEEVDPEIADIIEHEKARQWKGLELIPSENFTSVSVMQAVGSVMTNKYSE
Danio            -------------MLTLTLRQITRPLCRRVQRSSAAVCVRADGSWTGQESLSQDDPEMWDLLLKEKDRQCRGLELIASENFCSRAALEAQGSCLNNKYSE
Mus              --MVSFSLLRTTRPLQRCGQLVCMAARAQHSKVAQTQAGEATGGWTGQESLSDSDPEMWELLQREKDRQCRGLELIASENFCSRAALEALGSCLNNKYSE
Naegleria        --MQTTKRSVSVVTSSLKRTLASGQKRNFSSGIVNNGVNTEKWAAQLNASLKDVDPELFDIIEHEKNRQYKGLQLIPSENFTSKAVLDALGSVMQNKYSE
Thermosinus      -----------------------------------------------MNVLAGIDPEIAQAIDLERQRQQNKLELIASENFVSKAVMEAQGSVLTNKYAE
Geobacter        -----------------------------------------------MSVLETFDPAVAEVIRQETERQEYNLELIASENFVSPAVLEAQGSVLTNKYAE
Thermosipho      ----------------------------------------------MWENVKKTDPEIYDVILKEWERQEYGLELIASENFASLAVIEAMGSVLTNKYAE
Deferribacter    --------------------------------------------MSLYEAVKNIDPQVYDALMKELNRQETHIELIASENFVSKAVLEAQGSIMTNKYAE
Pelosinus        -----------------------------------------------MIKLKEFDPELAEAIELEKNRQQNKLELIASENFVTPMVMEAMGTVLTNKYAE
Hippea           -----------------------------------------------MSVLKDFDPDVYQAIENEKKRQMYGLELIASENLVSEAVLEAQGSIMTNKYAE
```

## Ornithine transcarbamylase

```
Trimastix       MSAPVATPTRTSSRQALRNAKALIAKIRKLNP-ALFEKDFLRTWDKSQDDIQTVLLVAQVLEELVKENIDTRVFHGGVAVSNFRDNSTRTRFSFQFAASL
Ruminococcus    ----------------MKTLQDYIDKLNSLNFKEMYNNDFFWTWDKTDDELEAVFTVADALRFMRENNISTKVFESGLGISIFRDNSTRTRFSFASACNL
Escherichia     ---------------MMKTVNELIKDINSLTS-HLHEKDFLLTWEQTPDELKQVLDVAAALKALRAENISTKVFNSGLGISVFRDNSTRTRFSYASALNL
Anaerococcus    ---------------MTQDIKKLIDELQELDHEKLFGNDFFHTWDKSQDELKAIWKTADILRAMREENISPKIFDSGLGISLFRDNSTRTRFSFASACNL
Klebsiella      ----------------MKTVNELIKDINKLNS-HLSEKDFLLTWEQSPDELKQVLDVAAALKTLRAENIATKVFNSGLGISVFRDNSTRTRFSYASALNL
Vibrio          ----------------MKNMNELIKEISQLES-KLHDKDFLLTWEQSPEELERVLKTAQVLKTMRGENIATNVFKNGLGISLFRDNSTRTRFSYASAINM
Yokenella       ----------------MKTVNELIKDINTLNS-HLHEKDFLLTWEQSPDELKQVLDVAAALKALRAENVATNVFNNGLGISVFRDNSTRTRFSYASALNL
Sebaldella      -------------MDKMKQFEEIIARLEKLDYKDMLNDDFFLTWDKSTDELNSVFAVADALRNLREQNISTKVFDSGLGISLFRDNSTRTRFSFASACNL
```

## Cpn60

```
Trimastix        --------------MQALFSTAAPALVRLASSAKEIKFGDEGRAAILKGVDTLAKAVATTLGPKGRTVLID-----DEF-EPRITKDGVT
Arabidopsis      MYRVLSKLSSSIGSSTSRKLVSGRIISSRNYAAKDISFGIGARAAMLQGVSEVAEAVKVTMGPKGRNVIIE-----SSYGGPKITKDGVT
Saccharomyces    ---------MLRSSVVRSRATLRPLLRRAYSSHKELKFGVEGRASLLKGVETLAEAVAATLGPKGRNVLIE-----QPFGPPKITKDGVT
Nyctotherus      ------MRGLTNFVSKQATRSLISTPMRAIGTGKDISFGIEARKSLLDGCDKLADAVQLTLGPKGRNVVLD-----KGYGIPKITKDGVT
Trichomonas      ----------------MSLIEAAKHFTRAFAKARDLKFGSDARDHLLLGVEKLADAVVSTLGPKGRNVMIE-----LPYGPPKVTKDGVT
Cryptosporidium  ---------------------MTSVVPKIMDDGAQESKGELARLQSIVGAIAIGDLLKTTFGPKGMDKILQPIKEGPIDSTPIVTNDGAT
Entamoeba        -----------------------MLSSSSHYNGKLLSLNIDCRENVLSGIKKVADAVSVTLGPKGRTVIID-----QPYGNARVTKDGVS
Giardia          ----------------------------MLQHYTSVISGEDARSGLLRGIKTIADVVATTLGPRGRAVILADG---SASGTTKVTKDGVS
Ehrlichia        -------------------------------MANMVVTGEQLDKSIREVVRILEDAVGCTAGPKGLTVAIS-----KPYGAPEVTKDGYK
Brucella         ------------------------------MAAKDVKFGRTAREKMLRGVDILADAVKVTLGPKGRNVVIE-----KSFGAPRITKDGVS
```

## H-protein of GCS

```
Trimastix        ----------------------------MQRLFSVVPAVGLSFLARFAGEKFYTKDHEWVDES----GLVGISDYAQKHLGQIVYVDLPE
Homo             MALRVVRSVRALLCTLRAVPSPAAPCPPRPWQLGVGAVRTLRTGPALLSVRKFTEKHEWVTTENG-IGTVGISNFAQEALGDVVYCSLPE
Dictyostelium    --------------------------MLKTLRFGTRAFGQNLNIAKRNFCTRYTNDHEWVTSLGSQNYRLGITDFAQKQLGDIVFVEIPQ
Arabidopsis      -------------------MACRLFWASRVASHLRISVAQRGFSSVVLKDLKYADSHEWVKIDGN-KATFGITDHAQDHLGDVVYVELPD
Trypanosoma      -----------------------------MRRALFCPAATVAATIRFYTTRFFTDSHEWVEHGDG-IATIGITAHAQENLGDVVYVALPN
Trichomonas      -------------------------------------MISTLCNCSRNFTKLYAKTHEYIDVEGT-IGKIGLSEFAMKMLGKATFVDV-Q
Vibrio           ----------------------------------------------MESTLKFTSSHEWIRDNGDGTVTVGISDHAQGLLGDVVFVDLPE
Bartonella       -----------------------------------------------MSKVYFTQDHEWLNIEGE-IATVGITHYAQEQLGDLVFVDLPQ
Acidiphilium     -----------------------------------------------MTETRFSKDHEWVRLDGD-VATVGITDHAQSALGDVVFVELPE
```

## P1-protein of GCS

```
Trimastix        ---------------------------------------------------------------MQNLSRAAHTASDILPYVAHTPEVRAQ
Solanum          MERARKLANRAILKRLVSQSKQSRSNEIPSSSLYRPSRYVSSLSPYTFQARNNAKSFNTQQARSISVEALKPSDTFPRRHNSATPEEQTK
Mus              --------MQLCARAWGLRLGRGAGGGHRLARGTGLSWAQRSRDSSGGGGGGGGGDRGAAGASRLLERLLPRHDDFSRRHIGPGDKDRRE
Saccharomyces    ---------MLRTRVTALLCRATVRSSTNYVSLARTRSFHSQSILLKTAATDITSTQYSRIFNPDLKNIDRPLDTFARRHLGPSPSDVKK
Paramecium       ---------------------------------------MLRSIRLIKRVANFSRLANPHEVNPKIPDWFKSSDYIESRFIGSEPQQVKE
Trypanosoma      ---------------------------------------------------MYRSAFRSLRCSTVKYARWSAAGNYVNRHVGPTAADTEV
Pyrococcus       ---------------------------------------------------------------------------MAKHYIPN-SAHKEE
Legionella       -----------------------------------------------------------------------------MPYIPHTPNDTKE
Desulfovibrio    -----------------------------------------------------------------------------MPYTPHTPEEIRQ
Xanthobacter     -----------------------------------------------------------------------------MRYLPLTPEDRAE
```

## T-protein of GCS

```
Trimastix        ------------------------MLSKLLSLPTTGILERFSSHVATKKTPFYDLHMKFGGDVTDFCGYYLPIKYANS--DIGIEHMNTR
Dictyostelium    ------------------MNTSGLFLKSLKASTRASNKCFFSSSTELKKTSLNQLHKELGGKMVPFCGWEMPVQYPA---GVLKEHMHCR
Chlorella        MRRQAAVQLLRALGRAGSDAAASAKHSNSCAAAGLLATRGFADDASLKKTVLYDYHVANGGKMVPFAGWSMPIQYKD---SIIDSTLHCR
Capsaspora       ------------MLCRSATAVAASIVARSAARSTAVRCGSTAATGPSKRTALYDFHVAKGGKMVPFAGWDMPVQYSDL--GIIASHHHTR
Thalassiosira    -----------MLKSTATALLRRAKRTSILPSTSSRSLASSTNEEPLVKTSLYNLHKELGGDMVPFAGYELPVLYKGDNGGVMKEHLWCR
Vibrio           ------------------------------------------MTQELLKTPLHALHVEAGAKMVPFAGYDMPVQYKL---GVKKEHLHTR
Parvibaculum     ------------------------------------MSEPAVQTEILKTTPLHALHVELGAKMVPFAGYDMPVQYPD---GVLAEHLHTR
Halomonas        -------------------------------------------MSELKQTPLHALHLKLGAKMVPFAGYDMPVQYPL---GVKKEHEHTR
Oceanospirillum  -------------------------------------------MSELSKTPLYDLHVELGGKMVPFAGFEMPVQYPL---GVKKEHVHTR
```
