# Supplementary material for: The Mitochondrion-Like Organelle of Trimastix pyriformis Contains the Complete Glycine Cleavage System
Source: PLoS One. 2013 Mar 13;8(3):e55417. doi: 10.1371/journal.pone.0055417 (PMC3596361; doi:10.1371/journal.pone.0055417)

Figure S2. Immunofluorescence microscopy of two additional *Trimastix pyriformis* cells. The green signal from antiH-protein (human) co-localizes with red signal from the antiH-protein (*Trimastix*). The DNA is stained blue with Hoechst.

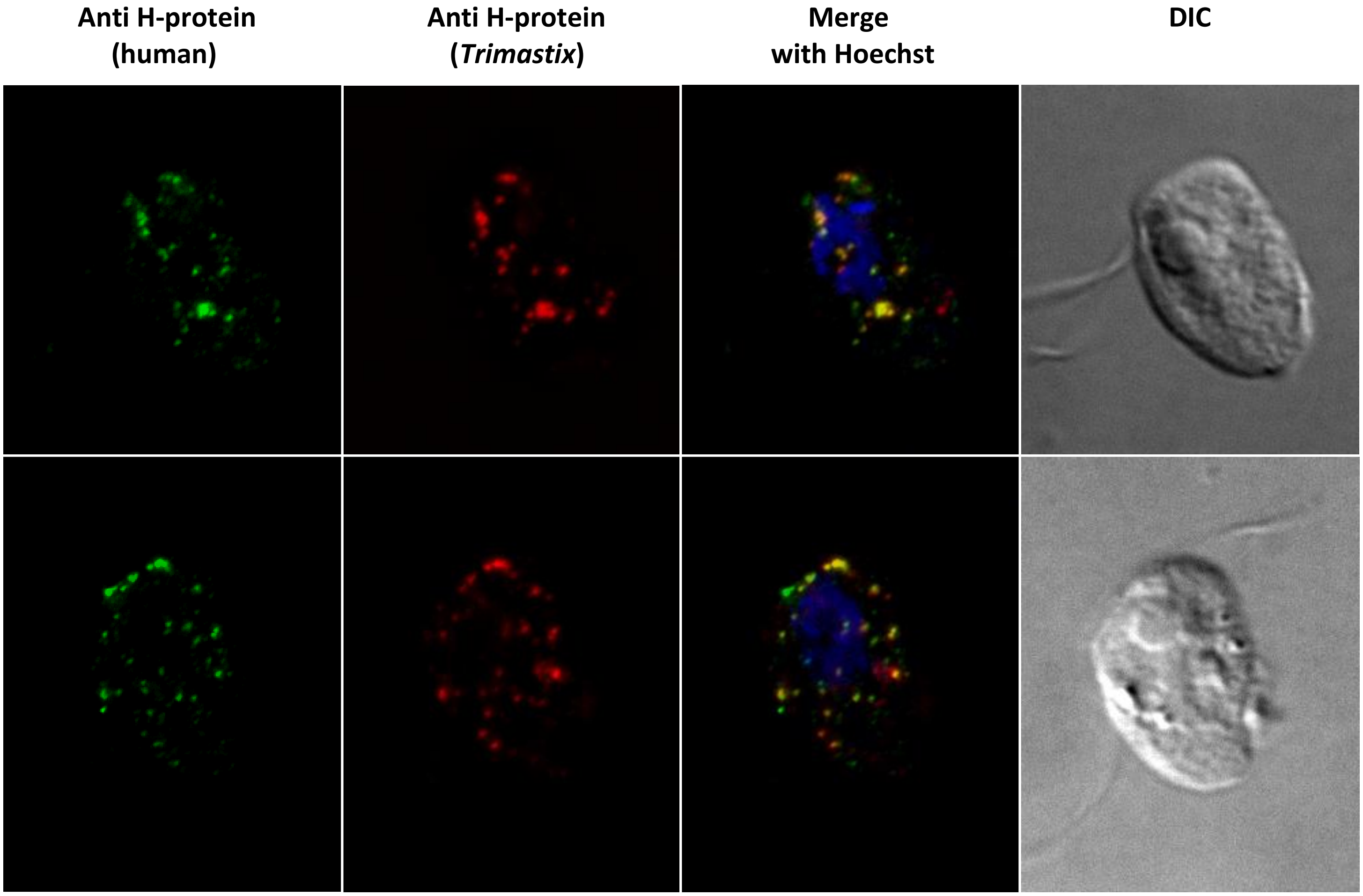

Supplement: Figure S2 — Immunofluorescence microscopy of two additional Trimastix pyriformis cells. The green signal from antiH-protein (human) co-localizes with red signal from the antiH-protein (Trimastix). The DNA is stained blue with Hoechst. (PDF) [file pone.0055417.s002.pdf]
