# Supplementary material for: The Mitochondrion-Like Organelle of Trimastix pyriformis Contains the Complete Glycine Cleavage System
Source: PLoS One. 2013 Mar 13;8(3):e55417. doi: 10.1371/journal.pone.0055417 (PMC3596361; doi:10.1371/journal.pone.0055417)
Supplement: Table S1 — The probability of mitochondrial localization of selected Trimastix proteins as predicted by PSORT II, TargetP and Multiloc2 programs. (DOCX) [file pone.0055417.s003.docx]

**Table S1:** The probability of mitochondrial localization of selected *Trimastix* proteins as predicted by PSORT II [1], TargetP [2] and Multiloc2 [3] programs.

| **Protein** | **PSORT II** | **TargetP** | **MultiLoc2** |
| --- | --- | --- | --- |
| **aconitase** | ND | 0.194 | 0.3 |
| **HydE** | 0.174 | 0.767 | 0.92 |
| **HydF** | 0.609 | 0.708 | 0.35 |
| **H-protein** | 0.348 | 0.186 | 0.33 |
| **P1-protein** | 0.174 | 0.269 | 0.2 |
| **T-protein** | 0.13 | 0.305 | 0.94 |
| **SHMT** | 0.87 | 0.083 | 0.42 |
| **OTC** | 0.522 | 0.733 | 0.84 |
| **aMPP** | 0.261 | 0.170 | 0.18 |
| **Cpn60** | 0.174 | 0.167 | 0.04 |

**REFERENCES**

1. Nakai, K. and Horton, P., PSORT: a program for detecting the sorting signals of proteins and predicting their subcellular localization, *Trends Biochem. Sci,* **24**(1) 34-35 (1999).
2. Locating proteins in the cell using TargetP, SignalP, and related tools Olof Emanuelsson, Søren Brunak, Gunnar von Heijne, Henrik Nielsen *Nature Protocols* 2, 953-971 (2007)
3. Blum, T, Briesemeister, S, and Kohlbacher, O (2009) MultiLoc2: integrating phylogeny and Gene Ontology terms improves subcellular protein localization prediction BMC Bioinformatics, 10:274.
